# Association of Baseline Prostate-Specific Antigen Level With Long-term Diagnosis of Clinically Significant Prostate Cancer Among Patients Aged 55 to 60 Years: A Secondary Analysis of a Cohort in the Prostate, Lung, Colorectal, and Ovarian (PLCO) Cancer Screening Trial

**DOI:** 10.1001/jamanetworkopen.2019.19284

**Published:** 2020-01-15

**Authors:** Evan Kovac, Sigrid V. Carlsson, Hans Lilja, Jonas Hugosson, Michael W. Kattan, Erik Holmberg, Andrew J. Stephenson

**Affiliations:** 1 Glickman Urological and Kidney Institute, Cleveland Clinic Foundation, Cleveland, Ohio; 2 Department of Urology, Montefiore Medical Center, Bronx, New York; 3 Department of Epidemiology and Biostatistics, Memorial Sloan Kettering Cancer Center, New York, New York; 4 Urology Service, Department of Surgery, Memorial Sloan Kettering Cancer Center, New York, New York; 5 Department of Urology, Institute of Clinical Sciences, Sahlgrenska Academy at University of Gothenburg, Sweden; 6 Department of Laboratory Medicine, Memorial Sloan Kettering Cancer Center, New York, New York; 7 Department of Medicine, Memorial Sloan Kettering Cancer Center, New York, New York; 8 Nuffield Department of Surgical Sciences, University of Oxford, Oxford, United Kingdom; 9 Department of Translational Medicine, Lund University, Malmö, Sweden; 10 Department of Quantitative Health Sciences, Cleveland Clinic Foundation, Cleveland, Ohio; 11 Department of Oncology, Institute of Clinical Sciences, Sahlgrenska Academy at University of Gothenburg, Sweden; 12 Department of Surgery, Division of Urology, Rush Medical College, Chicago, Illinois

## Abstract

**Importance:**

The use of prostate-specific antigen (PSA) screening for prostate cancer is controversial because of the risk of overdiagnosis and overtreatment of indolent cancers. Optimal screening strategies are highly sought.

**Objective:**

To estimate the long-term risk of any prostate cancer and clinically significant prostate cancer based on baseline PSA levels among men aged 55 to 60 years.

**Design, Setting, and Participants:**

This secondary analysis of a cohort in the Prostate, Lung, Colorectal, and Ovarian (PLCO) Cancer Screening Trial uses actuarial analysis to analyze the association of baseline PSA levels with long-term risk of any prostate cancer and of clinically significant prostate cancer among men aged 55 to 60 years enrolled in the screening group of the trial between 1993 and 2001.

**Exposure:**

Single PSA measurement at study entry.

**Main Outcomes and Measures:**

Long-term risk of any prostate cancer and clinically significant prostate cancer diagnoses.

**Results:**

There were 10 968 men aged 55 to 60 years (median [interquartile range] age, 57 [55-58] years) at study enrollment in the screening group of the PLCO Cancer Screening Trial who had long-term follow-up. Actuarial 13-year incidences of clinically significant prostate cancer diagnosis among participants with a baseline PSA of 0.49 ng/mL or less was 0.4% (95% CI, 0%-0.8%); 0.50-0.99 ng/mL, 1.5% (95% CI, 1.1%-1.9%); 1.00-1.99 ng/mL, 5.4% (95% CI, 4.4%-6.4%); 2.00-2.99 ng/mL, 10.6% (95% CI, 8.3%-12.9%); 3.00-3.99 ng/mL, 15.3% (95% CI, 11.4%-19.2%); and 4.00 ng/mL and greater, 29.5% (95% CI, 24.2%-34.8%) (all pairwise log-rank *P* ≤ .004). Only 15 prostate cancer–specific deaths occurred during 13 years of follow-up, and 9 (60.0%) were among men with a baseline PSA level of 2.00 ng/mL or higher.

**Conclusions and Relevance:**

In this secondary analysis of a cohort from the PLCO Cancer Screening Trial, baseline PSA levels among men aged 55 to 60 years were associated with long-term risk of clinically significant prostate cancer. These findings suggest that repeated screening can be less frequent among men aged 55 to 60 years with a low baseline PSA level (ie, <2.00 ng/mL) and possibly discontinued among those with baseline PSA levels of less than 1.00 ng/mL.

## Key Points

**Question:**

Are baseline prostate-specific antigen levels in midlife associated with future prostate cancer and clinically significant prostate cancer diagnoses?

**Findings:**

In this secondary analysis of a cohort of 10 968 men aged 55 to 60 years who were enrolled in the screening group of the Prostate, Lung, Colorectal, and Ovarian Cancer Screening Trial, baseline prostate-specific antigen levels were associated with any future prostate cancer and clinically significant prostate cancer diagnoses. The risk was significantly lower among men with a baseline prostate-specific antigen level of less than 2.00 ng/mL.

**Meaning:**

Future prostate cancer screening among middle-aged men (ie, aged 55-60 years) should be individually tailored based on baseline PSA levels to reduce the risk of overdiagnosis and overtreatment of indolent cancers.

## Teaser

This secondary analysis of the Prostate, Lung, Colorectal, and Ovarian (PLCO) Cancer Screening Trial estimates the long-term risk of any prostate cancer and clinically significant prostate cancer associated with baseline prostate-specific antigen (PSA) levels among men aged 55 to 60 years.

## Introduction

Since the introduction of widespread prostate-specific antigen (PSA) screening in the early 1990s, diagnosis and radical treatment of prostate cancer (PCa) has led to a 50% reduction in PCa-specific mortality since peak rates.^1^ While PSA screening was once widely accepted and ubiquitous, opinions on the utility of screening have shifted in recent years, and there is now controversy surrounding its use as a screening tool because of the overdiagnosis and overtreatment of indolent cancers.^2^ The concern over the utility of PSA as a screening tool was highlighted by the United States Preventive Services Task Force recommendation against PSA screening in 2012,^3^ although recently the task force has revised its statement to promote shared decision-making between patients and physicians.^4^ This position has factored in the increased use of active surveillance for men with low-risk PCa, as defined by the American Urological Association.^5,6^

Until a more accurate biomarker is identified, PSA remains the best screening tool available for early PCa diagnosis. Therefore, it is imperative to improve the detection of clinically significant PCa while minimizing overdiagnosis and overtreatment through a more nuanced approach to PSA screening.

Previous reports have demonstrated that baseline PSA levels in young men are associated with PCa diagnosis later in life.^7,8,9,10,11^ However, few studies have analyzed baseline PSA levels on a large scale, and studies have seldom differentiated indolent PCa diagnoses from clinically significant PCa diagnoses as a function of baseline PSA.^12^

The Prostate, Lung, Colorectal, and Ovarian (PLCO) Cancer Screening Trial was a large, multicenter trial that randomized more than 76 000 men aged 55 to 74 years to receive either organized PSA screening or usual care with PCa-specific mortality as its end point.^13,14^ The trial did not demonstrate any statistically significant difference in PCa mortality between trial groups after 17 years of follow-up,^15^ likely because of high PSA screening contamination in the control group.^16,17^ However, meticulous data capture and extended follow-up provide valuable insights on patients in the screening group. In this secondary analysis, we sought to determine whether baseline PSA levels in younger men enrolled in the PLCO Cancer Screening Trial were associated with the long-term risk of any PCa diagnosis and clinically significant PCa diagnosis.

## Methods

Data from 13 years of follow-up from the PLCO Cancer Screening Trial were collected in a prospective, centrally maintained database and were used for this study. Centralized institutional review board approval was granted for this study, and written informed consent was obtained at study entry. This study follows the Strengthening the Reporting of Observational Studies in Epidemiology (STROBE) reporting guideline for cohort studies.

We limited our analysis to younger patients (ie, age 55-60 years at study enrollment) from the screening group who enrolled from 1993 to 2001 and received at least 1 PSA test. Information on baseline demographic characteristics, PSA levels at study enrollment, diagnostic prostate biopsy Gleason grade results, clinical staging, and PCa-specific mortality were prospectively captured. For men who underwent radical prostatectomy, pathological Gleason grade, pathological staging, and lymph node status were obtained. The definition of clinically significant PCa was based on findings from biopsy^18,19^ and radical prostatectomy specimen^20,21^ and was directly linked to the cancer’s propensity to metastasize and cause PCa-specific mortality.^22,23^ Therefore, based on updated guidelines, we defined clinically significant PCa as clinical stage of cT2b or greater, biopsy Gleason grade of 7 or greater, or PCa-specific mortality. Among patients who underwent radical prostatectomy, we defined clinically significant PCa as pathologic stage of pT3 or greater, pathologic Gleason grade of 7 of greater, or node-positive disease.^24^ For patients who underwent radical prostatectomy and for whom there was a discrepancy between clinical and pathologic Gleason grade, we used the highest Gleason grade to define clinically significant PCa.

### Statistical Analysis

Using nonparametric univariate analysis, we estimated the 13-year rates (with 95% CIs) of any PCa diagnosis and clinically significant PCa diagnosis among younger patients (ie, aged 55-60 years at study enrollment) as a function of baseline PSA. Patients were divided into the following clinically relevant baseline PSA groups: PSA level of 0.49 ng/mL or less, 0.50 to 0.99 ng/mL, 1.00 to 1.99 ng/mL, 2.00 to 2.99 ng/mL, 3.00 to 3.99 ng/mL, and 4.00 ng/mL or greater (to convert to μg/L, multiply by 1.0). The Kaplan-Meier method was used to generate any PCa and clinically significant PCa incidence curves over time, and differences between baseline PSA groups were tested using the log-rank method.

Competing risks regression analyses were used for modeling risk of any PCa and clinically significant PCa. Deaths from other causes were treated as competing risks. Baseline PSA as a continuous variable was the only factor, and restricted cubic splines were used to relax the linear association of baseline PSA with the outcomes.

Statistical significance was set at *P* ≤ .05. All statistical tests were 2-tailed. Statistical analyses were performed using SPSS statistical software version 23 (IBM Corp) and R Core Team 2013 (R Project for Statistical Computing).

## Results

We identified 10 968 patients from the screening group of the PLCO Cancer Screening Trial who were aged 55 to 60 years at study enrollment and underwent PSA screening. Most men (9102 [83.0%]) had baseline PSA levels of less than 2.00 ng/mL. Overall, the 13-year Kaplan-Meier rate of any PCa and clinically significant PCa were 10.4% (95% CI, 9.8%-11.0%) and 4.8% (95% CI, 4.4%-5.2%), respectively. The median (interquartile range [IQR]) time from study enrollment to PCa diagnosis was 5.9 (2.6-9.3) years. The median (IQR) follow-up for the entire cohort until PCa diagnosis or end of study was 11.7 (10.1-12.8) years. The median (IQR) age and baseline PSA for the entire cohort were 57 (55-58) years and 0.95 (0.60-1.60) ng/mL, respectively. Baseline demographic and clinical characteristics of patients diagnosed with any PCa and clinically significant PCa are presented in Table 1.

**Table 1.** Baseline Characteristics of Patients Aged 55 to 60 Years and Diagnosed With Any PCa or Clinically Significant PCa From the Screening Group of the PLCO Cancer Screening Trial

Baseline Characteristic	PCa Diagnosis, No. (%)	
	Any (n = 970)	Clinically Significant (n = 425)
Age, median (IQR), y		
At study enrollment	57 (55-58)	57 (55-58)
At PCa diagnosis	63 (59-66)	63 (60-66)
Baseline PSA level, median (IQR), ng/mL	2.4 (1.5-3.8)	2.3 (1.5-3.9)
Baseline PSA level group, No./Total No. (%)		
≤0.49 ng/mL	13/1792 (0.7)	5/1792 (0.3)
0.50-0.99 ng/mL	88/3936 (2.2)	43/3936 (1.1)
1.00-1.99 ng/mL	291/3374 (8.6)	135/3374 (4.0)
2.00-2.99 ng/mL	205/973 (21.1)	83/973 (8.5)
3.00-3.99 ng/mL	148/442 (33.5)	55/442 (12.4)
≥4.00 ng/mL	225/451 (49.9)	104/451 (23.1)
Clinical T stage		
1	659 (67.9)	257 (60.5)
2	295 (30.4)	157 (36.9)
3	10 (1.0)	10 (2.4)
Missing	6 (0.6)	1 (0.2)
Clinical N stage		
0	967 (99.7)	424 (99.8)
1	3 (0.3)	1 (0.2)
Clinical M stage		
0	958 (98.7)	415 (97.6)
1	12 (1.2)	10 (2.4)
Biopsy Gleason score		
≤6	667 (68.8)	156 (36.7)
7	229 (23.6)	207 (48.7)
8	37 (3.8)	36 (8.5)
9	23 (2.3)	23 (5.4)
10	3 (0.3)	3 (0.7)
Missing	11 (1.1)	0
Pathologic Gleason Score		
Total	570 (100)	303 (100)
≤6	287 (50.4)	24 (7.9)
7	232 (40.7)	237 (78.2)
8	21 (3.7)	21 (6.9)
9	19 (3.3)	19 (6.3)
10	2 (0.4)	2 (0.7)
Missing	9 (1.6)	0

Abbreviations: IQR, interquartile range; PCa, prostate cancer; PLCO, Prostate, Lung, Colorectal, and Ovarian; PSA, prostate-specific antigen.

SI conversion: To convert PSA to μg/L, multiply by 1.0.

The median (IQR) age at PCa diagnosis was 63 (59-66) years. Overall 13-year Kaplan-Meier primary PCa treatment rate was 9.5% (95% CI, 8.9%-10.1%), and the 13-year Kaplan-Meier radical prostatectomy rate was 6.0% (95% CI, 5.4%-6.6%).

Actuarial diagnosis of any PCa and clinically significant PCa, stratified by baseline PSA at 13 years follow-up, is presented in Table 2. Men with a baseline PSA level of 0.49 ng/mL or less had the lowest 13-year actuarial risk of any PCa (0.8%; 95% CI, 0.4%-1.2%) and clinically significant PCa (0.4%; 95% CI, 0%-0.8%). Men with a baseline PSA level of 0.50 to 0.99 ng/mL and 1.00 to 1.99 ng/mL also had a low actuarial risk of long-term PCa and clinically significant PCa diagnosis. Specifically, younger men (ie, aged 55-60 years) with a baseline PSA level between 0.50 and 0.99 ng/mL had an estimated 1.5% (95% CI, 1.1%-1.9%) long-term incidence of clinically significant PCa, and for men with a baseline PSA level between 1.00 and 1.99 ng/mL, the actuarial rate was 5.4% (95% CI, 4.4%-6.4%). Compared with men with a baseline PSA level between 1.00 and 1.99 ng/mL, the 13-year risk of clinically significant PCa nearly doubled for men with a baseline PSA level between 2.00 and 2.99 ng/mL (10.6%; 95% CI, 8.3%-12.9%). Among men with a baseline PSA level between 3.00 and 3.99 ng/mL, the 13-year risk of clinically significant PCa was 15.3% (95% CI, 11.4%-19.2%). The 13-year risk of any PCa and clinically significant PCa incidence was highest among men with a baseline PSA level of 4.00 ng/mL or greater (any PCa: 53.7%; 95% CI, 48.6%-58.8%; clinically significant PCa: 29.5%; 95% CI, 24.2%-34.8%).

**Table 2.** 13-Year Actuarial Rates of Any PCa Diagnosis and Clinically Significant PCa Diagnosis Based on Baseline PSA Measurements in Patients Aged 55 to 60 Years Enrolled in the Screening Group of the PLCO Cancer Screening Trial

Baseline PSA, ng/ml	13-Year Cumulative Incidence, % (95% CI)	
	Any PCa Diagnosis	Clinically Significant PCa Diagnosis
≤0.49	0.8 (0.4-1.2)	0.4 (0-0.8)
0.50-0.99	3.2 (2.2-4.2)	1.5 (1.1-1.9)
1.00-1.99	11.2 (9.8-12.6)	5.4 (4.4-6.4)
2.00-2.99	24.0 (21.1-26.9)	10.6 (8.3-12.9)
3.00-3.99	36.9 (31.8-42.0)	15.3 (11.4-19.2)
≥4.00	53.7 (48.6-58.8)	29.5 (24.2-34.8)

Abbreviations: PCa, prostate cancer; PLCO, Prostate, Lung, Colorectal, and Ovarian; PSA, prostate-specific antigen.

SI conversion: To convert PSA to μg/L, multiply by 1.0.

**Figure 1.**
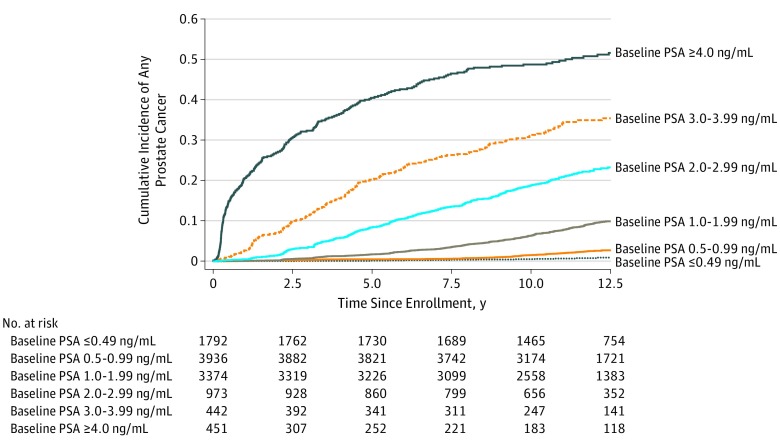
13-Year Kaplan-Meier Risk of Any Prostate Cancer Among Patients Aged 55 to 60 Years Enrolled in the Screening Group of the PLCO Cancer Screening Trial, Stratified by Baseline Prostate-Specific Antigen (PSA) Level To convert PSA to micrograms per liter, multiply by 1.0. PLCO indicates Prostate, Lung, Colorectal, and Ovarian.

**Figure 2.**
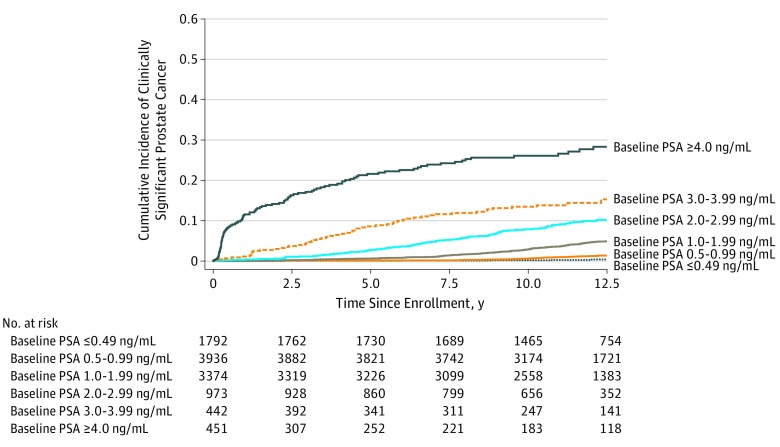
13-Year Kaplan-Meier Risk of Clinically Significant Prostate Cancer Among Patients Aged 55 to 60 Years Enrolled in the Screening Arm of the PLCO Cancer Screening Trial, Stratified by Baseline Prostate-Specific Antigen (PSA) Level To convert PSA to micrograms per liter, multiply by 1.0. PLCO indicates Prostate, Lung, Colorectal, and Ovarian.

Figure 1 and Figure 2 depict Kaplan-Meier curves of any PCa diagnosis and clinically significant PCa diagnosis, respectively, during the 13-year study period for patients aged 55 to 60 years, stratified by baseline PSA group. All log-rank pairwise comparisons of baseline PSA groups met statistical significance at *P* ≤ .004.

The 13-year probabilities of any PCa and clinically significant PCa over different baseline PSA levels and adjusted for competing risk events are depicted in eFigure 1 and eFigure 2, respectively, in the Supplement. The knots of the restrictive cubic spline were placed at quartiles of the distribution of baseline PSA level (ie, 0.59, 0.92, and 1.48 ng/mL).

Overall, 893 patients died during the study period; only 15 of these deaths were PCa-specific, and 9 (60.0%) were among men with baseline PSA levels of 2.00 ng/mL or higher. Because of the small number of PCa-specific mortality events, we did not perform an actuarial analysis of PCa-specific mortality based on baseline PSA level.

## Discussion

Among men entering the screening group of the PLCO Cancer Screening Trial at age 55 to 60 years, the risk of being diagnosed with a clinically significant PCa during a median of 11.7 years of follow-up (and an actuarial follow-up of 13 years) differed significantly among those in different baseline PSA level groups and was lowest among men with a baseline PSA level of 0.49 ng/mL or lower. In addition, while our results suggest that there is no absolute baseline PSA cutoff level under which the 13-year risk of any PCa or clinically significant PCa is 0, this risk was higher among men with a baseline PSA level of 2.00 ng/mL or greater.

At a time when the concern regarding the overdiagnosis and overtreatment of indolent PCa has led to intense scrutiny of the value of PSA levels as an effective screening tool,^3,4^ our data on the increasing risks associated with higher baseline PSA levels can be used to determine the optimal PCa screening strategy for individual patients. The low 13-year actuarial risk of a clinically significant PCa diagnosis in middle-aged men with a baseline PSA level less than 2.00 ng/mL (ie, 0.4% among men with PSA levels <0.49 ng/mL, 1.5% among men with PSA levels of 0.50-0.99 ng/mL, and 5.4% among men with PSA levels of 1.00-1.99 ng/mL) suggests that men younger than 60 years with PSA levels of 1.00 to 1.99 ng/mL can undergo less intensive screening than men with higher baseline PSA levels. Men with baseline PSA levels less than 1.00 ng/mL may wish to discontinue screening. Most men would be affected by these changes in screening strategy because 83% of men in our study had baseline PSA levels less than 2.00 ng/mL.

In contrast, men with baseline PSA levels of 2.00 to 2.99 ng/mL, 3.00 to 3.99 ng/mL, and especially 4.00 ng/mL or greater had 13-year actuarial risks of a clinically significant PCa diagnosis of 10.6%, 15.3%, and 29.5%, respectively. These men are likely to benefit from more intensive screening strategies.

A total of 3 large randomized trials (ie, the PLCO Cancer Screening Trial,^15^ the European Randomized Study of Screening for Prostate Cancer,^25^ and the Göteborg trial^26^) sought to determine whether organized PSA screening prevents PCa-specific mortality. While the 2 European trials (ie, the European Randomized Study of Screening for Prostate Cancer^25^ and the Göteborg trial^26^) concluded that organized PSA screening led to a significant reduction in PCa-specific mortality (HRs 0.80 and 0.65, respectively), the PLCO Cancer Screening Trial did not show a difference in PCa-specific mortality between the screening and control groups. However, the conflicting results of the PLCO Cancer Screening Trial are likely attributable to high rates of contamination in the control group and low biopsy adherence rates, thus masking the potential benefit of PSA screening in the trial.^16,17^ Taken together, we conclude that, while PSA screening is likely beneficial, more data are needed to determine the optimal screening interval and the PSA level cutoff for screening cessation, based on the man’s age and general health status. This report furthers the empirical evidence to help define such risk-stratified algorithms.

Previous reports have examined the association of baseline PSA level among younger men with any future PCa diagnosis^9^ as well as risk of PCa metastasis and PCa-specific mortality.^12^ Other studies^27^ have reported on the association of baseline PSA level with clinically significant PCa in high-risk groups, such as men with African ancestry. However, these prior studies either had relatively small cohort sizes or analyzed very homogenous populations, unlike that of the United States. For example, a retrospective analysis of frozen plasma samples in men enrolled in the Malmö Diet and Cancer study^28^ found a strong association between baseline PSA level, other kallikrein markers, and PCa-specific mortality after 20 years of follow-up. However, the subcohort was small (ie, 1223 PCa events and 3028 controls) compared with our cohort. In addition, commercially available 4-kallikrein tests are relatively expensive and not routinely used in primary care practices, whereas a single, baseline PSA level test in midlife is relatively inexpensive and offers significant and comparable prognostic information.

Therefore, our study is among the largest series to date to analyze the association of a single PSA level, taken in midlife, with the long-term diagnosis of clinically significant PCa among men in the United States. We believe that this contemporary subset analysis of younger men enrolled in the screening group of the PLCO Cancer Screening Trial strengthens the merits of early baseline PSA level screening.^29,30^ Longer follow-up of the PLCO Cancer Screening Trial to 20 years, expected in 2022, will hopefully provide much-anticipated PCa-specific mortality data associated with baseline PSA testing.

Within the last 2 decades, the distinction between low-risk, indolent PCa and clinically significant PCa that has metastatic potential and may cause PCa-specific morbidity and mortality has been recognized. Not all prostate cancers are biologically aggressive, and improvements in selective screening practices and risk-stratification tools are highly sought so that only individuals with a significant risk of harboring clinically significant PCa undergo prostate biopsy.

We found that the 13-year incidence of clinically significant PCa diagnosis (as defined by those with metastatic potential,^22,23^ ie, clinical or pathologic Gleason grade, ≥7; clinical stage, ≥cT2b; pathologic stage, ≥pT3; pathologic node-positive disease; or death from PCa) among men aged 55 to 60 years with PSA levels less than 2.00 ng/mL ranged from 0% to 5%, while the 13-year risk among similarly aged men with PSA levels of 2.00 ng/mL or greater ranged from 11% to 30%.

Of note, overall and clinically significant PCa diagnosis rates were higher among men with PSA levels of 4.00 ng/mL or greater during the early portion of the study period, and diagnoses were delayed among men with PSA levels less than 2.00 ng/mL (Figure 1 and Figure 2). This is likely owing to immediate biopsy and diagnosis among patients with higher and more suspicious PSA levels at study entry vs delayed biopsy and diagnosis among patients with low PSA levels at study enrollment whose PSA levels slowly increased to suspicious levels during the study period.

There are several potential clinical interpretations of these results. First, our results suggest that younger men with a low baseline PSA level can be screened less frequently without significantly increasing the risk of future PCa-specific morbidity or mortality. Second, improved risk-stratification of screened patients could lead to a reduction in the number of PSA tests and unnecessary prostate biopsies, thus reducing the rates of biopsy-related sepsis, anxiety and stress among patients, overdiagnoses, and overall costs to the health care system.

In addition to baseline PSA levels, several novel reflex biomarkers have been validated to determine the risk of harboring aggressive PCa on biopsy^31,32,33^ in men with elevated serum PSA levels and/or abnormal digital rectal examinations and may help reduce the need for prostate biopsy. However, these biomarkers are costly and not widely available. Baseline PSA level is attractive as a biomarker because PSA testing is already widely available, relatively inexpensive, and involves only a paradigm shift in PCa screening intensity and possible cessation of future PCa screening based on baseline PSA level measured between the ages of 55 and 60 years.

### Limitations

The study has limitations. Our analysis is a post hoc analysis from a large, randomized clinical trial and lends itself to inherent biases. The actuarial follow-up period of 13 years is relatively short and few PCa-specific deaths occurred in our study population during the study period—likely owing, in part, to the lead-time bias of PSA screening on PCa-specific mortality and the treatment of diagnosed clinically significant PCa. However, previous randomized clinical trials and observational studies have shown limited benefit of radical treatment for men older than 65 years diagnosed with localized PCa.^34,35^ Furthermore, while we cannot draw direct conclusions regarding the association of baseline PSA level with PCa-specific mortality in this study, we believe men aged 55 to 60 years who undergo baseline PSA testing and do not develop clinically significant PCa after 13 years of follow-up are unlikely to experience PCa-specific mortality. This supports our conclusion regarding the cessation of screening for those with a baseline PSA level less than 1.00 ng/mL.

Second, we analyzed long-term, PCa-specific outcomes among patients based on a single PSA value taken between the ages of 55 and 60 years, but we did not analyze other PSA metrics, such as PSA density or velocity. However, previous studies^36,37^ have shown that a single PSA level measurement, taken earlier in life, is more tightly associated with future PCa diagnosis and PCa-specific mortality than PSA velocity. We did not analyze the number of PSA tests taken subsequent to baseline PSA at study enrollment, which may have varied among baseline PSA groups. Additionally, biopsy adherence in the PLCO Cancer Screening Trial was not per protocol and was generally low,^38^ leading to ascertainment bias. Furthermore, while our cohort consisted of men enrolled in the PLCO Cancer Screening Trial at the age of 55 to 60 years, we believe that previous work has provided population-based evidence for a younger target age for baseline PSA testing,^30,39^ which is currently being prospectively evaluated in the randomized ProBase trial, enrolling men at age 45 to 50 years for baseline PSA testing.^40^

## Conclusions

To our knowledge, our analysis of baseline PSA in men aged 55 to 60 years who were enrolled in the screening group of the PLCO Cancer Screening Trial represents the largest analysis of its kind to date. Our results support the modification of future screening practices based on baseline PSA level and could help to reduce the need for prostate biopsy and overdetection of clinically indolent PCa without significantly compromising oncologic outcomes.

## Notes

Supplement. **eFigure 1.** Thirteen-Year Probability of Any PCa by Baseline PSA Level, Adjusting for Competing Risk Events, Among Patients Aged 55 to 60 Years and Enrolled in the Screening Arm of the Prostate, Lung, Colorectal, and Ovarian Cancer Screening Trial **eFigure 2.** Thirteen-Year Probability of Clinically Significant PCa by Baseline PSA Levels, Adjusting for Competing Risk Events, Among Patients Aged 55 to 60 Years and Enrolled in the Screening Arm of Prostate, Lung, Colorectal, and Ovarian Cancer Screening Trial
