# Supplementary material for: Association of Baseline Prostate-Specific Antigen Level With Long-term Diagnosis of Clinically Significant Prostate Cancer Among Patients Aged 55 to 60 Years: A Secondary Analysis of a Cohort in the Prostate, Lung, Colorectal, and Ovarian (PLCO) Cancer Screening Trial
Source: JAMA Netw Open. 2020 Jan 15;3(1):e1919284. doi: 10.1001/jamanetworkopen.2019.19284 (PMC6991265; doi:10.1001/jamanetworkopen.2019.19284)
Supplement: Supplement. — eFigure 1. Thirteen-Year Probability of Any PCa by Baseline PSA Level, Adjusting for Competing Risk Events, Among Patients Aged 55 to 60 Years and Enrolled in the Screening Arm of the Prostate, Lung, Colorectal, and Ovarian Cancer Screening Trial eFigure 2. Thirteen-Year Probability of Clinically Significant PCa by Baseline PSA Levels, Adjusting for Competing Risk Events, Among Patients Aged 55 to 60 Years and Enrolled in the Screening Arm of Prostate, Lung, Colorectal, and Ovarian Cancer Screening Trial [file jamanetwopen-3-e1919284-s001.pdf]

Supplementary Online Content

Kovac E, Carlsson SV, Lilja H, et al. Association of baseline prostate-specific antigen level with long-term diagnosis of clinically significant prostate cancer among patients aged 55 to 60 years: a secondary analysis of a cohort in the Prostate, Lung, Colorectal, and Ovarian (PLCO) Cancer Screening Trial. *JAMA Netw Open*. 2020;3(1):e1919284. doi:10.1001/jamanetworkopen.2019.19284

eFigure 1. Thirteen-Year Probability of Any PCa By Baseline PSA Level, Adjusting for Competing Risk Events, Among Patients Aged 55 to 60 Years and Enrolled in the Screening Arm of the Prostate, Lung, Colorectal, and Ovarian Cancer Screening Trial

eFigure 2. Thirteen-Year Probability of Clinically Significant PCa By Baseline PSA Levels, Adjusting for Competing Risk Events, Among Patients Aged 55 to 60 Years and Enrolled in the Screening Arm of Prostate, Lung, Colorectal, and Ovarian Cancer Screening Trial

This supplementary material has been provided by the authors to give readers additional information about their work.

© 2020 Kovac E et al. *JAMA Network Open.*

eFigure 1. Thirteen-Year Probability of Any PCa By Baseline PSA Level, Adjusting for Competing Risk Events, Among Patients Aged 55 to 60 Years and Enrolled in the Screening Arm of the Prostate, Lung, Colorectal, and Ovarian Cancer Screening Trial

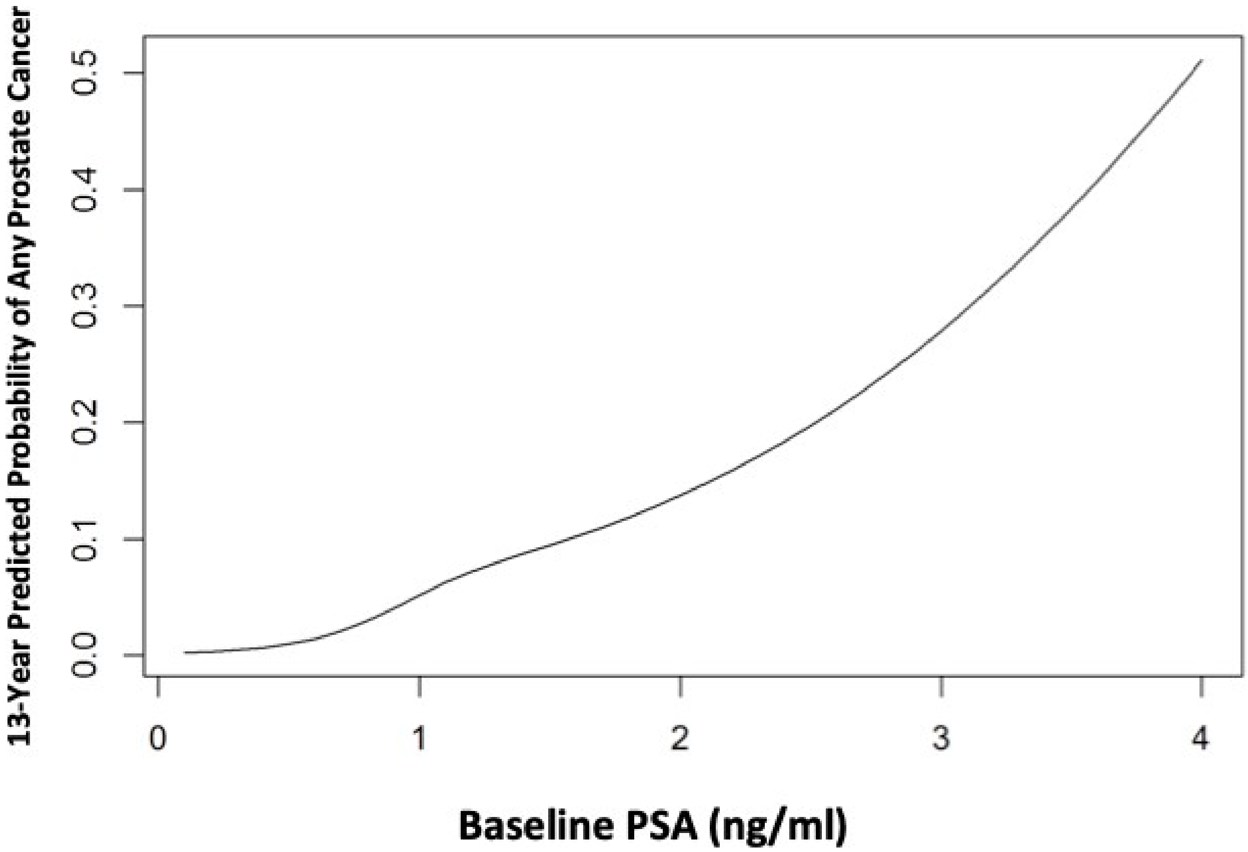

© 2020 Kovac E et al. *JAMA Network Open.*

eFigure 2. Thirteen-Year Probability of Clinically Significant PCa By Baseline PSA Levels, Adjusting for Competing Risk Events, Among Patients Aged 55 to 60 Years and Enrolled in the Screening Arm of Prostate, Lung, Colorectal, and Ovarian Cancer Screening Trial

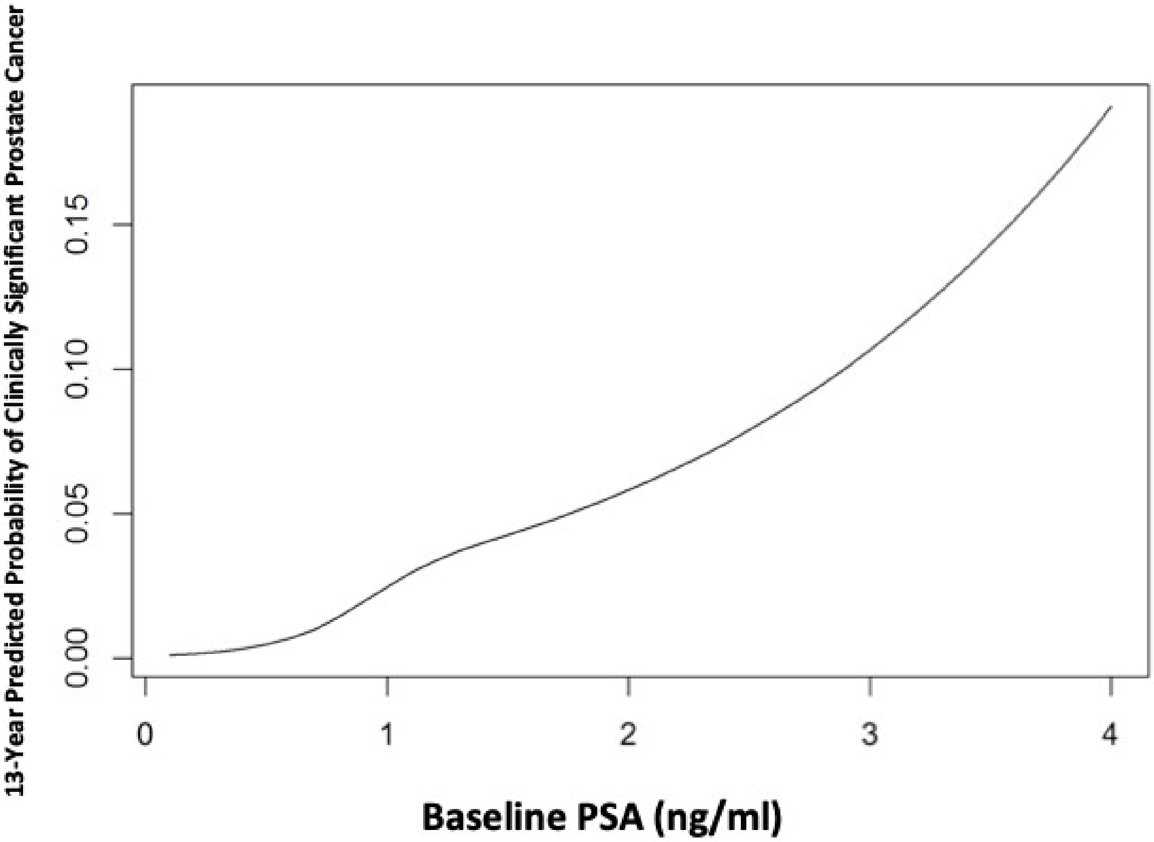

© 2020 Kovac E et al. *JAMA Network Open.*
